# Supplementary material for: Warming-induced vapor pressure deficit suppression of vegetation growth diminished in northern peatlands
Source: Nat Commun. 2023 Nov 30;14:7885. doi: 10.1038/s41467-023-42932-w (PMC10689446; doi:10.1038/s41467-023-42932-w)
Supplement: Supplementary file 1 — Supplementary Information [file 41467_2023_42932_MOESM1_ESM.pdf]

Supplementary Information

Warming-induced vapor pressure deficit suppression of vegetation growth diminished in northern peatlands

Ning Chen[1,2], Yifei Zhang[1], Fenghui Yuan[1,7], Changchun Song[1,3*], Mingjie Xu[5], Qingwei Wang[2], Guangyou Hao[2], Tao Bao[6], Yunjiang Zuo[1], Jianzhao Liu[1,8], Tao Zhang[5], Yanyu Song[1], Li Sun[1], Yuedong Guo[1], Hao Zhang[1], Guobao Ma[1], Yu Du[1], Xiaofeng Xu[4*], Xianwei Wang[1*]

[1] Key Laboratory of Wetland Ecology and Environment, Northeast Institute of Geography and Agroecology, Chinese Academy of Sciences, Changchun 130102, China.
[2] CAS Key Laboratory of Forest Ecology and Management, Institute of Applied Ecology, Chinese Academy of Sciences, Shenyang 110016, China.
[3] School of Hydraulic Engineering, Dalian University of Technology, Dalian, 116024, China.
[4] Biology Department, San Diego State University, San Diego, 92182, USA.
[5] College of Agronomy, Shenyang Agricultural University, Shenyang, 110866, China.
[6] Key Laboratory of Regional Climate-Environment for Temperate East Asia, Institute of Atmospheric Physics, Chinese Academy of Sciences, Beijing, China.
[7] Department of Soil, Water, and Climate, University of Minnesota, Saint Paul, MN, 55108, USA.
[8] College of Surveying and Exploration Engineering, Jilin Jianzhu University, Changchun 130018, China.

Corresponding author: Changchun Song (songcc@iga.ac.cn); Xiaofeng Xu (xxu@sdsu.edu); Xianwei Wang (wangxianwei@iga.ac.cn)

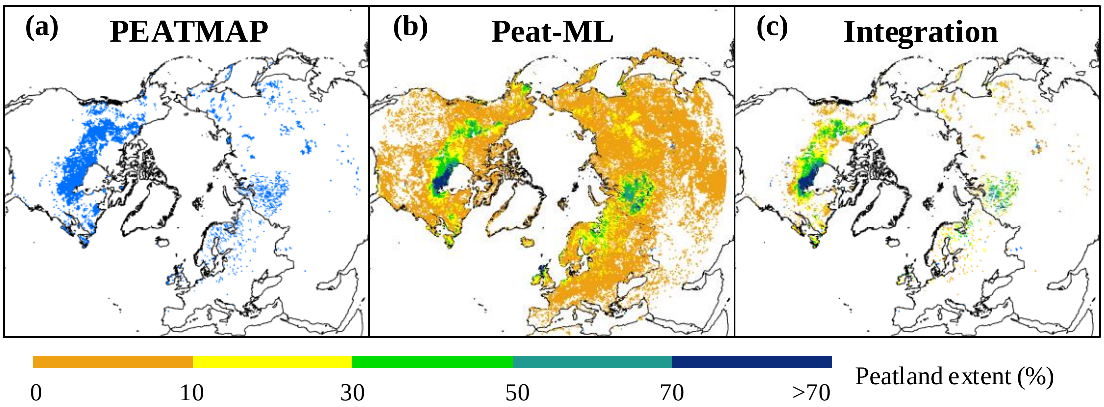

Supplementary Fig. 1. Spatial extent of northern peatland estimated by Peat-ML (a), PEATMAP (b), and their integration (c); peatland extent (%) infers the percentage of peatland in each grid.

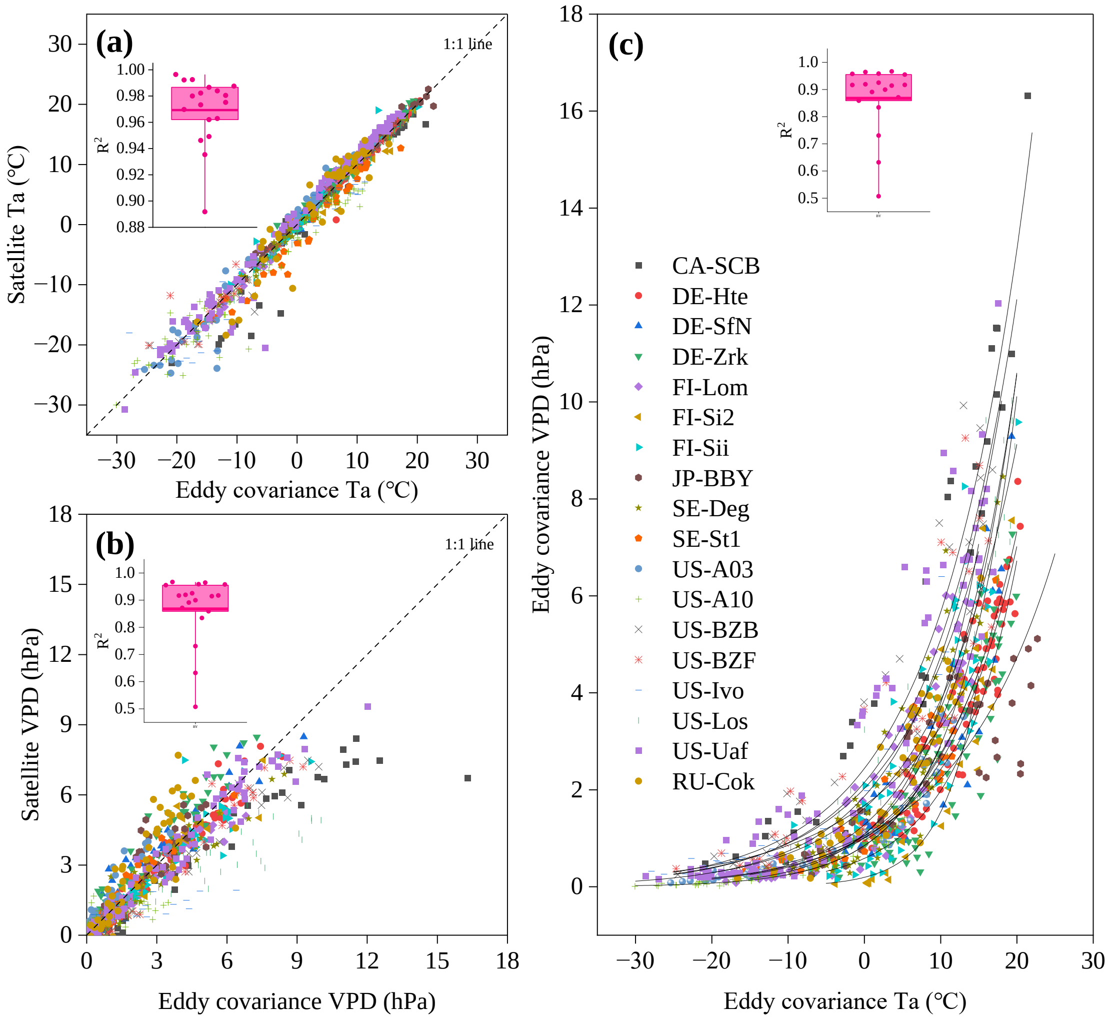

Supplementary Fig. 2. Correlations of satellite-derived air temperature (Ta) (vapor pressure deficit, VPD) with eddy-covariance Ta (VPD), and the exponential correlations of Ta with VPD based on eddy covariance flux towers. a. eddy variance Ta vs. satellite Ta; **b. eddy covariance VPD vs. satellite VPD; c.** eddy covariance Ta vs. eddy covariance VPD**;** Boxes indicate the statistical distributions of the correlation coefficients (R^2) of these relationships in 18 sites (dot). The lines in the boxes indicate the mean of R^2. The maximum and minimum extents of the colored boxes indicate the 25th and 75th percentiles, and the whiskers represent the 5th and 95th percentiles, respectively.

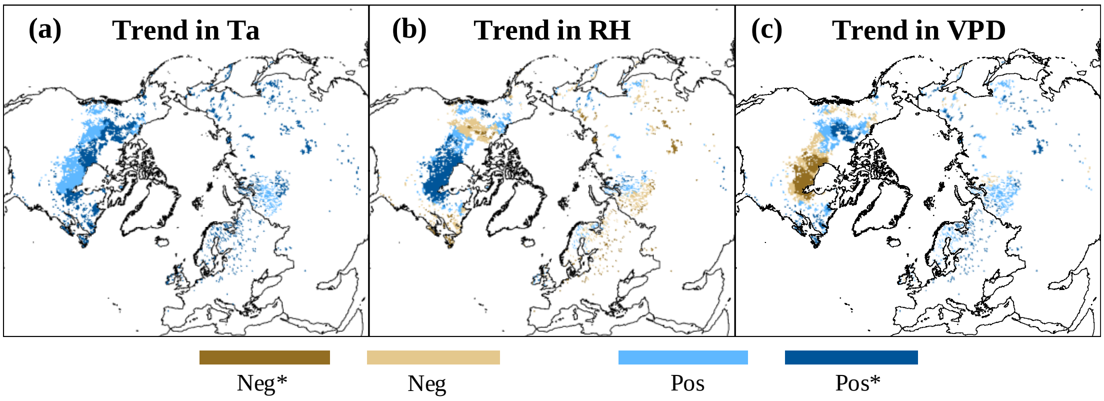

Supplementary Fig. 3. Spatial distributions of changing trends of (a) air temperature (Ta), (b) relative humidity (RH), and (c) vapor pressure deficit (VPD) from 1982 to 2018 in the northern peatlands. Significant (Neg*; $p < 0.05$; dark brown) and insignificant (Neg; $p > 0.05$; light brown) decreases in the rates of Ta, RH, and VPD; significant (Pos*; $p < 0.05$; dark blue) and insignificant (Pos; $p > 0.05$; light blue) increases in the rates of Ta, RH, and VPD.

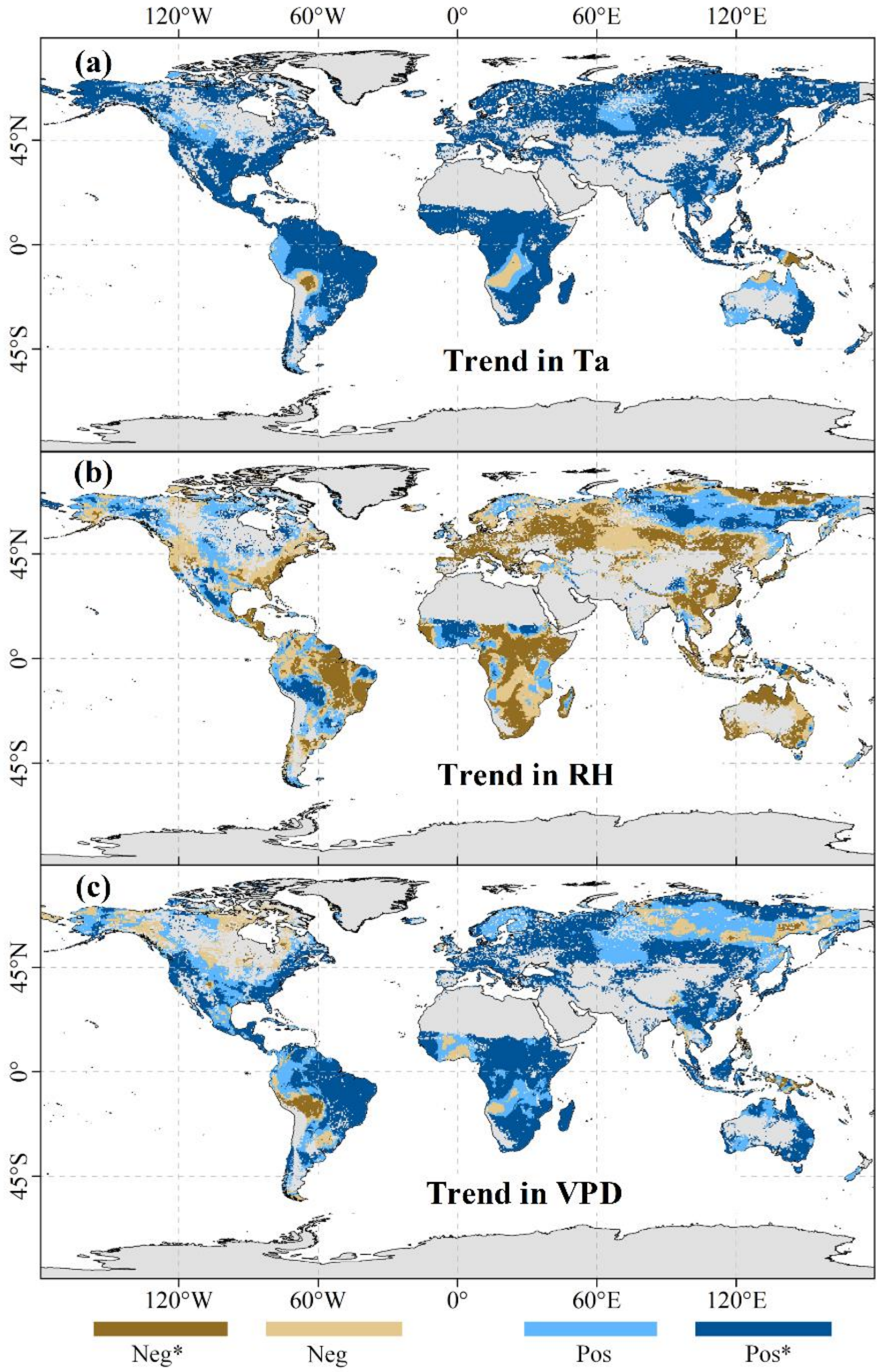

Supplementary Fig. 4. Spatial distributions of changing trends of (a) air temperature (Ta), (b) relative humidity (RH), and (c) vapor pressure deficit (VPD) from 1982 to 2018 in the global nonpeatland regions. Significant (Neg*; $p < 0.05$; dark brown) and insignificant (Neg; $p > 0.05$; light brown) decreases in the rates of Ta, RH, and VPD; significant (Pos*; $p < 0.05$; dark blue) and insignificant (Pos; $p > 0.05$; light blue) increases in the rates of Ta, RH, and VPD.

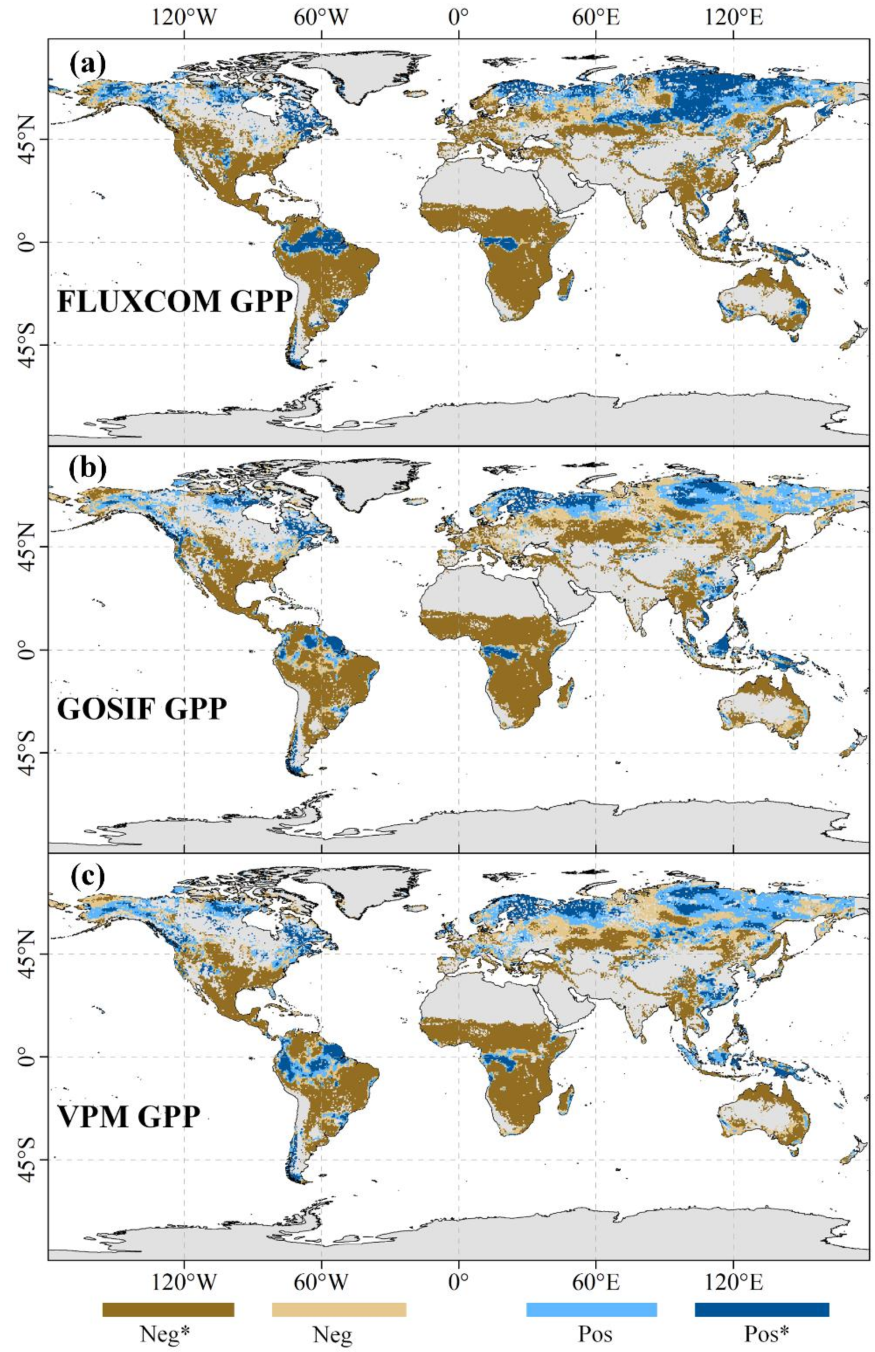

Supplementary Fig. 5. Spatial distributions of the impacts of VPD on GPP ((a) FLUXCOM GPP, (b) GOSIF GPP, (c) VPM GPP) in the global nonpeatland regions. Significant (Neg*; $p < 0.05$; dark brown) and insignificant (Neg; $p > 0.05$; light brown) negative correlations; significant (Pos*; $p < 0.05$; dark blue) and insignificant (Pos; $p > 0.05$; light blue) positive correlations.

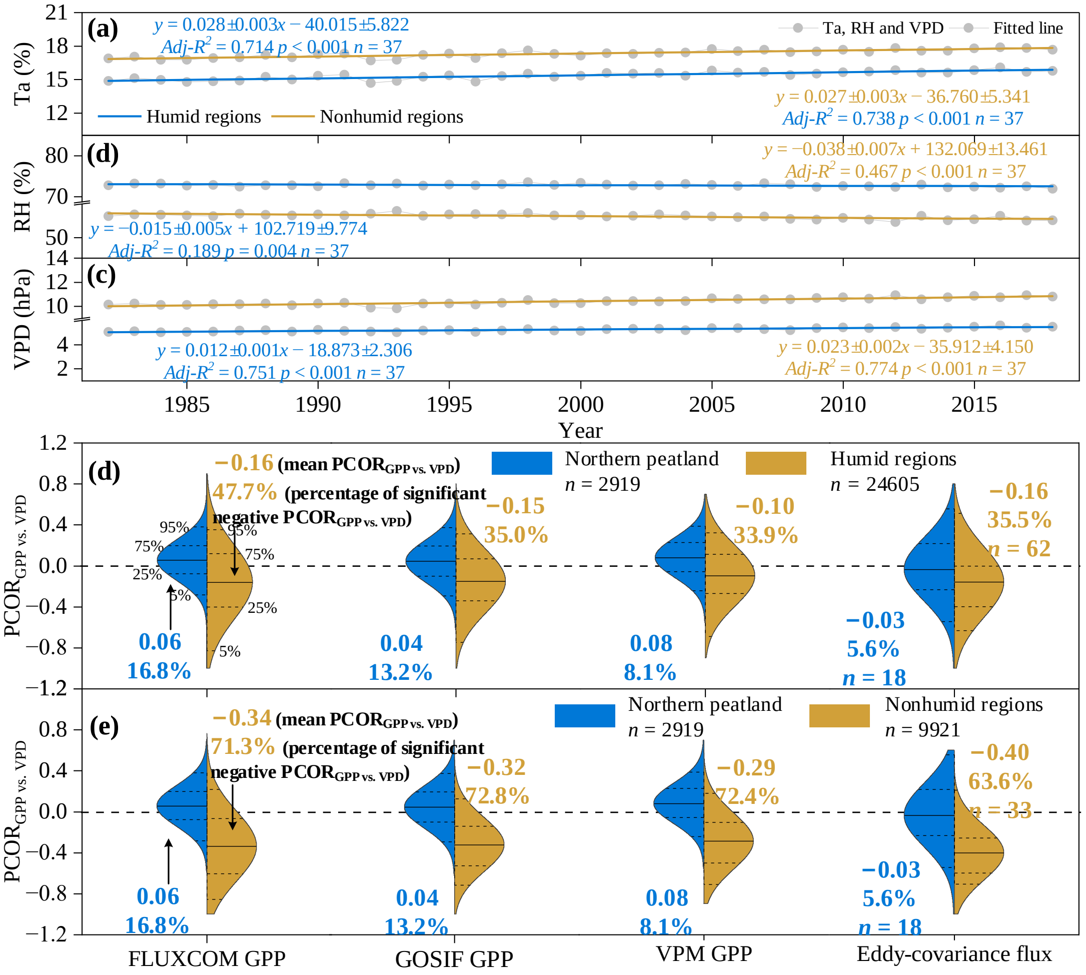

Supplementary Fig. 6. Temporal dynamics of air temperature (Ta), relative humidity (RH), and vapor pressure deficit (VPD) from 1982 to 2018, and VPD effects in the global nonpeatland regions of the humid regions and nonhumid regions. a−c: Time series of Ta, RH, and VPD from 1982 to 2018 in the humid regions (blue equations) and the nonhumid regions (brown equations). Adj-R^2: Adjusted R Square. **d−e**: Differences in the VPD effects as estimated by the mean $PCOR_{GPP\ vs.\ VPD}$ and the percentage (%) of significantly negative $PCOR_{GPP\ vs.\ VPD}$ between the northern peatlands (blue) and the humid regions (brown, d) and the nonhumid regions (brown, e). “*n*” (*n* = 2919 and *n* = 24605 indicate the satellite-derived GPP) indicates sample size. Solid lines in the violin plots indicate the mean $PCOR_{GPP\ vs.\ VPD}$. The dotted lines show the maximum (95th percentile) and minimum (5th percentile) extent and the 25th and 75th percentiles in the violin plots.

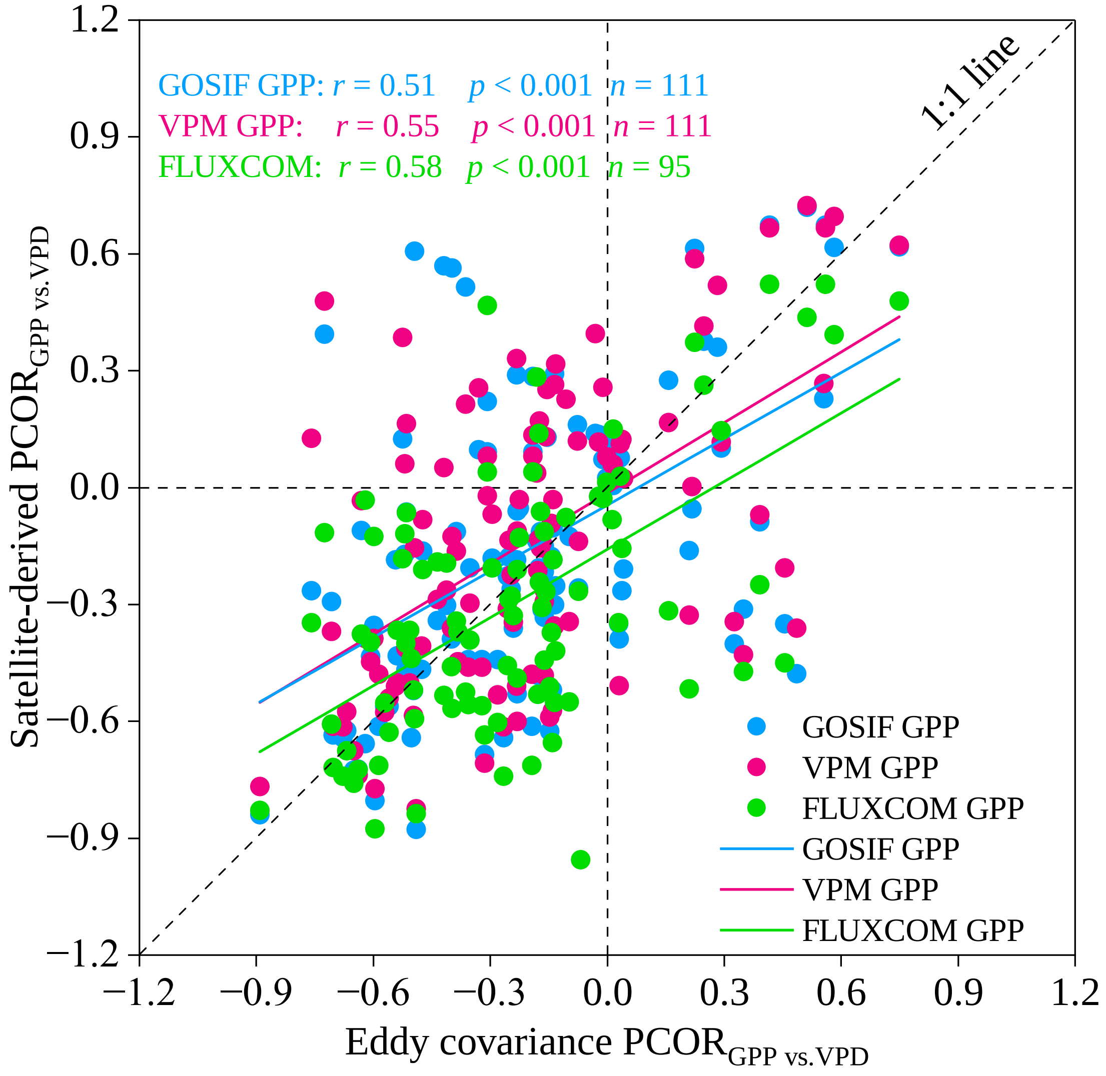

Supplementary Fig. 7. Correlations of eddy covariance $PCOR_{GPP\ vs.\ VPD}$ with satellite-derived $PCOR_{GPP\ vs.\ VPD}$ that estimated by FLUXCOM GPP, VPM GPP, and GOSIF GPP. "*r*" indicates the correlation coefficient. The statistical tests (*p*) for correlation coefficient are two-sided. "*n*" indicates sample size. $PCOR_{GPP\ vs.\ VPD}$ in the 1st and 3rd quadrants show the same symbols between eddy covariance $PCOR_{GPP\ vs.\ VPD}$ and satellite-derived $PCOR_{GPP\ vs.\ VPD}$, 2nd and 4th quadrants show the opposite symbols.

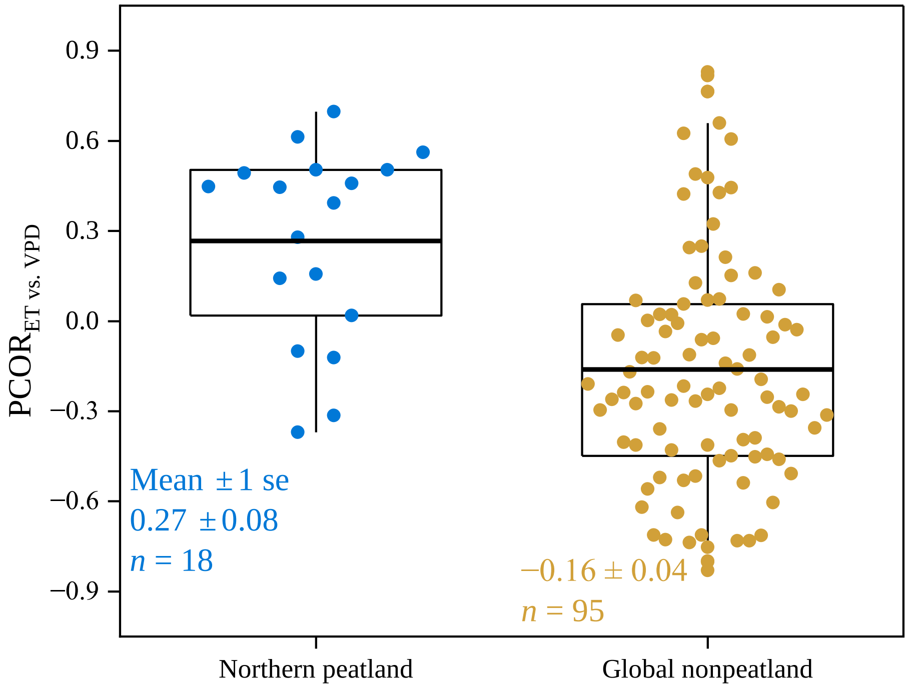

Supplementary Fig. 8. Differences in responses of evapotranspiration (ET) to vapor pressure deficit (VPD) between the northern peatlands and the global nonpeatland regions based on eddy covariance flux datasets. Solid lines in the boxes indicate the mean of partial correlation coefficients (PCOR$_{ET\ vs.\ VPD}$). Blue and brown dispersion points indicate the distribution of PCOR$_{ET\ vs.\ VPD}$ for the northern peatlands and the global nonpeatland regions. The maximum and minimum extents of the boxes indicate the 25th and 75th percentiles, and the whiskers represent the 5th and 95th percentiles, respectively. "*n*" indicates sample size.

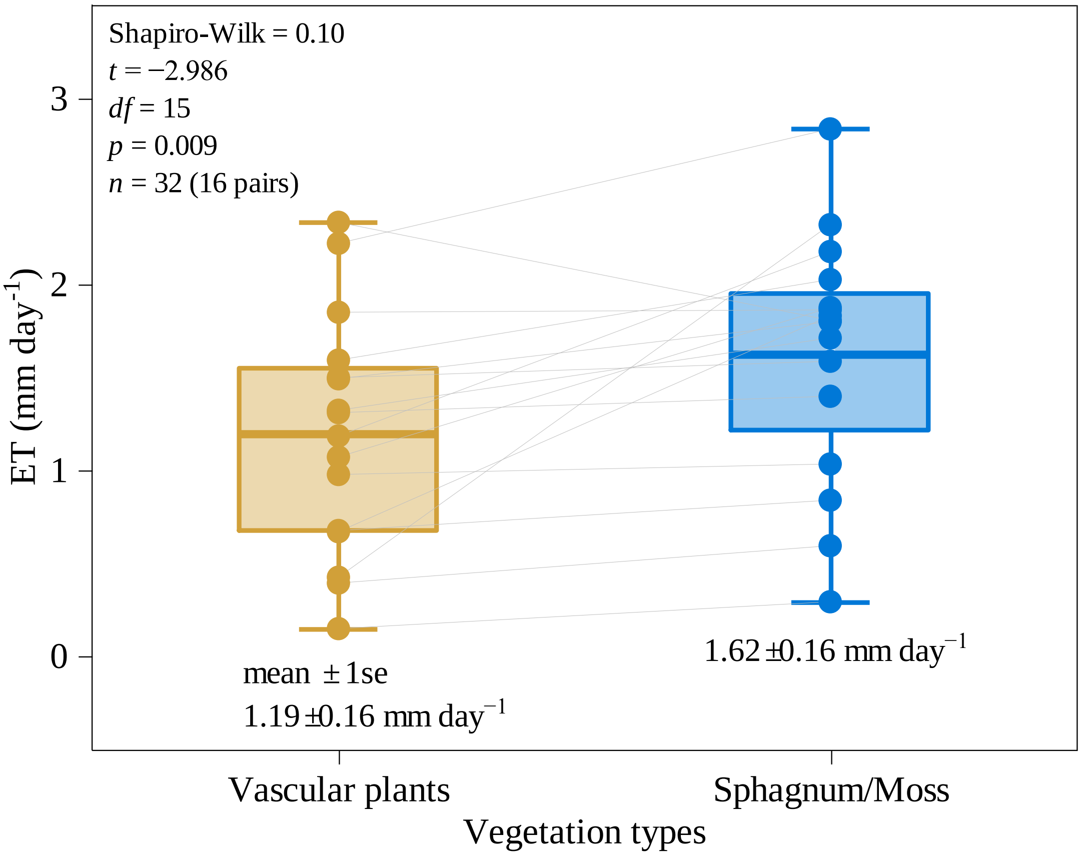

Supplementary Fig. 9. Differences in evapotranspiration (ET) between vascular plants and sphagnum/moss under the same environmental conditions. Grey lines show the pairwise comparisons of ET between vascular plants and sphagnum/moss. The lines in the boxes and the numbers at the bottom of the boxes show the mean ET of vascular plants and sphagnum/moss. The maximum and minimum extents of the boxes indicate the 25th and 75th percentiles, and the whiskers represent the 5th and 95th percentiles, respectively. "*n*" indicates sample size. Shapiro-Wilk is used to estimate the Tests of Normality. P-values, t-statistic, and degrees of freedom are outputs of paired t-tests of ET between vascular plants and sphagnum/moss under the same environmental conditions.

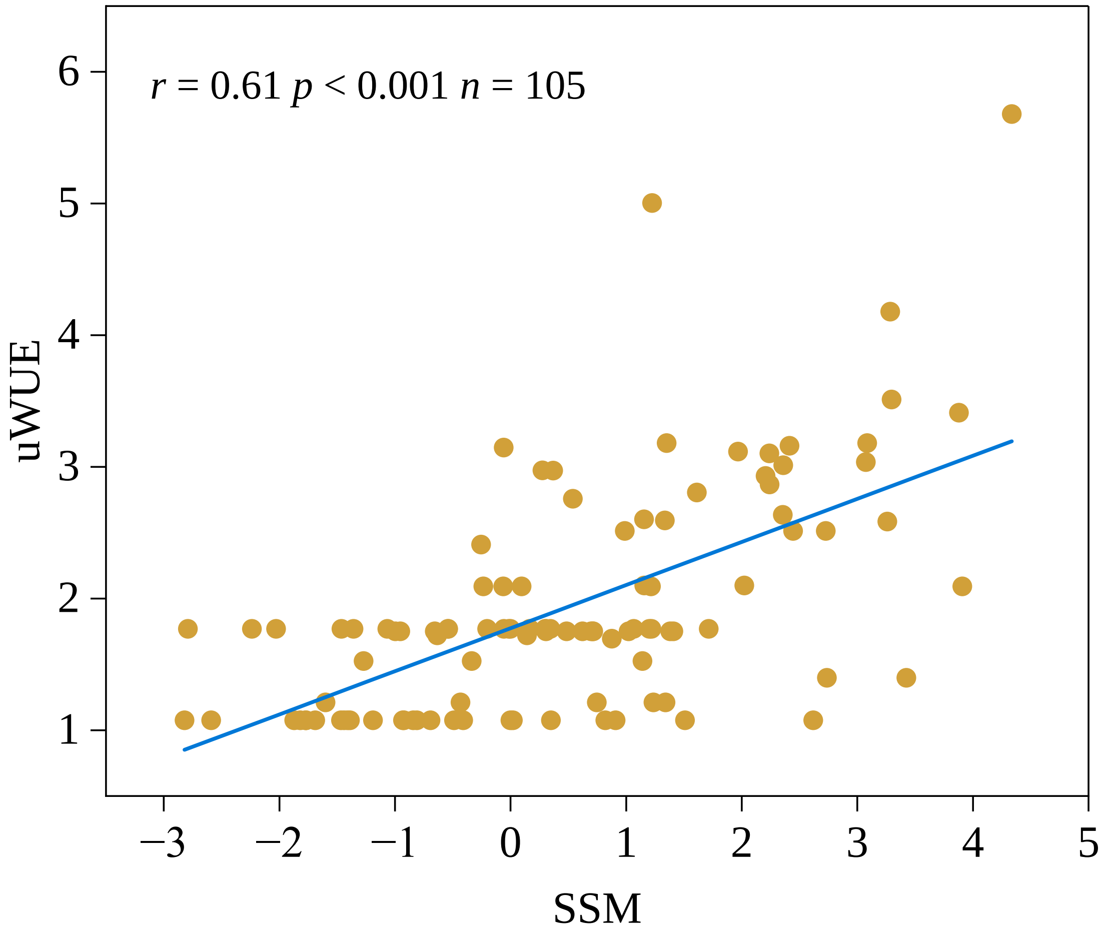

Supplementary Fig. 10. Correlations of underlying water use efficiency (uWUE) with stomatal safety margin (SSM). "*r*" indicates the correlation coefficient. The statistical tests (*p*) for correlation coefficient are two-sided. "*n*" indicates sample size.

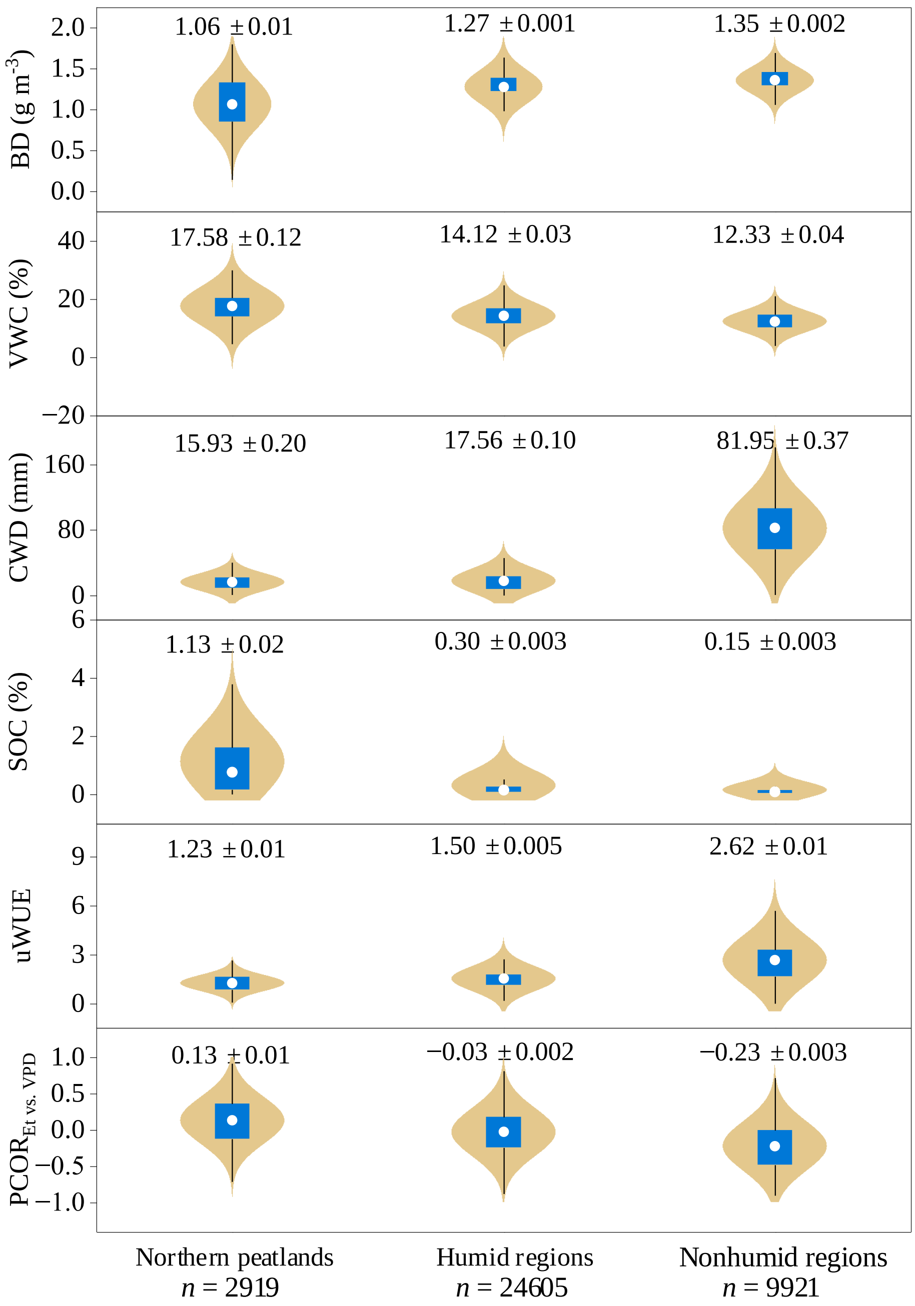

Supplementary Fig. 11. Differences in six plant traits and environmental factors between the northern peatlands and the humid regions, the nonhumid regions. White dots indicate arithmetic means. The numbers are the mean ± 1 standard error. BD: bulk density; VWC: available volumetric water content; CWD: climate water deficit; SOC: soil organic carbon; uWUE: underlying water use efficiency; $PCOR_{Et\ vs.\ VPD}$: transpiration (Et) response to VPD. The maximum and minimum extents of the boxes indicate the 25th and 75th percentiles, and the whiskers represent the 5th and 95th percentiles, respectively. “*n*” indicates sample size.

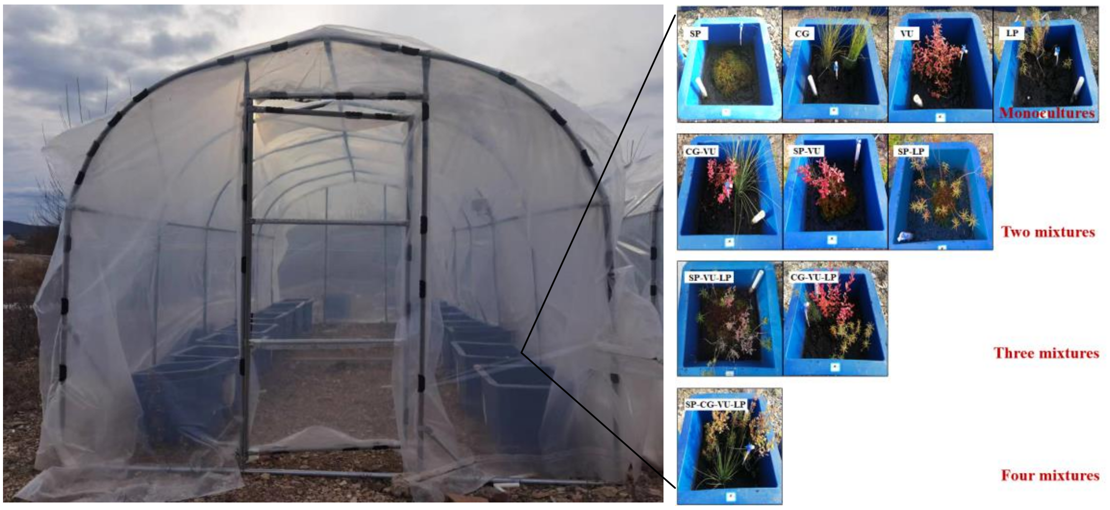

Supplementary Fig. 12. The warming experiment at the Mohe site. SP, CG, VU, and LP indicate *Sphagnum palustre*, *Carex globularis*, *Vaccinium uliginosum*, and *Ledum palustre*, respectively. Control and warming treatments included 60 mesocosms (square plastic barrels) (N = 30 per treatment), including four monocultures (SP, CG, VU, and LP), three mixtures of two species (CG-VU, SP-VU, and SP-LP), two mixtures of three species (SP-VU-LP and CG-VU-LP), and one mixture of four species (SP-CG-VU-LP) (N = 3 per species mixture). The warming treatment was placed in a transparent greenhouse and the control treatment was placed in an ambient environment near the transparent greenhouse.

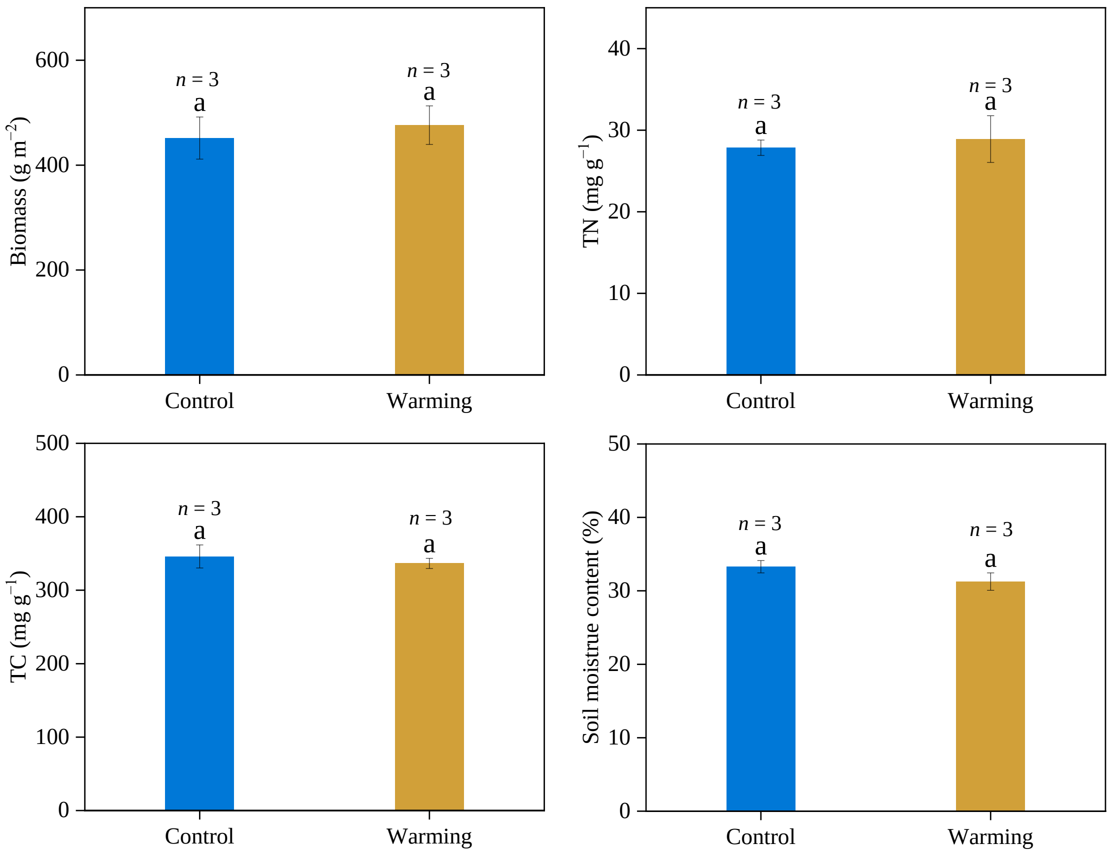

Supplementary Fig. 13 Biomass (mean ± 1 standard deviation), soil total carbon, soil total nitrogen, and soil moisture content under control and warming treatments at the Mohe site. Error bars represent one unit of standard deviation; same letters at the top of the histograms indicate insignificant differences between the control and warming treatments ($p > 0.05$).

Supplementary Table 1 Supporting information for synthesized 67 warming experiments to estimate the response of vegetation growth to increasing VPD in the northern peatlands, including geographical position, peatland type (Types), years of data collection (Time), exponential equations between Ta and VPD (Equation), and the correlation coefficients of the exponential equations (*r*).

ID	Latitude	Longitude	Types	Time	Equation	*r*
Boe_LT	68.63	-149.72	Tundra	1988-2001	$0.1594e^{0.0434x}$	0.91
Cha_G1	68.63	-149.57	Tundra	1981-1983	$0.1594e^{0.0434x}$	0.91
Cha_G2	68.63	-149.57	Tundra	1981-1989	$0.1594e^{0.0434x}$	0.91
Cha_L1	68.63	-149.57	Tundra	1981-1983	$0.1594e^{0.0434x}$	0.91
Cha_L2	68.63	-149.57	Tundra	1981-1989	$0.1594e^{0.0434x}$	0.91
Cha_M1	68.63	-149.57	Tundra	1981-1983	$0.1594e^{0.0434x}$	0.91
Cha_M2	68.63	-149.57	Tundra	1981-1989	$0.1594e^{0.0434x}$	0.91
Cha_S1	68.63	-149.57	Tundra	1981-1983	$0.1594e^{0.0434x}$	0.91
Cha_S2	68.63	-149.57	Tundra	1981-1989	$0.1594e^{0.0434x}$	0.91
Che_CN	34.72	92.88	Tundra	2014-2015	$0.4312e^{0.0586x}$	0.96
Dai_CQ	64.78	-64	Tundra	1995-1999	$0.0523e^{0.1101x}$	0.75
Dai_DA	64.78	-64	Tundra	1995-1999	$0.0523e^{0.1101x}$	0.75
Gro_TL	68.63	-149.57	Tundra	1997	$0.1594e^{0.0434x}$	0.91
Gro_TM	68.63	-149.57	Tundra	1997	$0.1594e^{0.0434x}$	0.91
Hob_G1	68.63	-149.57	Tundra	1990-1993	$0.1594e^{0.0434x}$	0.91
Hob_G2	68.63	-149.57	Tundra	1990-1993	$0.1594e^{0.0434x}$	0.91
Hob_S1	68.63	-149.57	Tundra	1990-1993	$0.1594e^{0.0434x}$	0.91
Hob_S2	68.63	-149.57	Tundra	1990-1993	$0.1594e^{0.0434x}$	0.91
Hob_S3	68.63	-149.57	Tundra	1990-1993	$0.1594e^{0.0434x}$	0.91
Hob_S4	68.63	-149.57	Tundra	1990-1993	$0.1594e^{0.0434x}$	0.91
Hob_S5	68.63	-149.57	Tundra	1990-1993	$0.1594e^{0.0434x}$	0.91
Hob_TL	68.63	-149.57	Tundra	1990-1993	$0.1594e^{0.0434x}$	0.91
Kle_MG	37.62	101.2	Tundra	1998-2001	$0.5026e^{0.0656x}$	0.96
Kle_MS	37.62	101.2	Tundra	1998-2001	$0.5026e^{0.0656x}$	0.96
Kle_SG	37.62	101.2	Tundra	1998-2001	$0.5026e^{0.0656x}$	0.96
Mol_AM	68.35	18.49	Tundra	1995-2006	$0.2229e^{0.0823x}$	0.84
Mol_DS	68.35	18.49	Tundra	1995-2006	$0.2229e^{0.0823x}$	0.84
Mol_ES	68.35	18.49	Tundra	1995-2006	$0.2229e^{0.0823x}$	0.84
Mol_GD	68.35	18.49	Tundra	1995-2006	$0.2229e^{0.0823x}$	0.84
Mol_LN	68.35	18.49	Tundra	1995-2006	$0.2229e^{0.0823x}$	0.84
Mol_PM	68.35	18.49	Tundra	1995-2006	$0.2229e^{0.0823x}$	0.84
Mol_SM	68.35	18.49	Tundra	1995-2006	$0.2229e^{0.0823x}$	0.84
Mun_KD1	55.27	-112.47	Bog	2011-2011	$0.104e^{0.1199x}$	0.91
Mun_KD2	55.27	-112.47	Bog	2011-2012	$0.104e^{0.1199x}$	0.91
Mun_KD3	55.27	-112.47	Bog	2011-2013	$0.104e^{0.1199x}$	0.91
Mun_KW1	55.35	-112.52	Bog	2011-2011	$0.104e^{0.1199x}$	0.91
Mun_KW2	55.35	-112.52	Bog	2011-2012	$0.104e^{0.1199x}$	0.91
Mun_KW3	55.35	-112.52	Bog	2011-2013	$0.104e^{0.1199x}$	0.91

Mun_WD1	55.27	-112.47	Bog	2011-2011	$0.104e^{0.1199x}$	0.91
Mun_WD2	55.27	-112.47	Bog	2011-2012	$0.104e^{0.1199x}$	0.91
Mun_WD3	55.27	-112.47	Bog	2011-2013	$0.104e^{0.1199x}$	0.91
Mun_WW1	55.35	-112.52	Bog	2011-2011	$0.104e^{0.1199x}$	0.91
Mun_WW2	55.35	-112.52	Bog	2011-2012	$0.104e^{0.1199x}$	0.91
Mun_WW3	55.35	-112.52	Bog	2011-2013	$0.104e^{0.1199x}$	0.91
Nat_A1	63.88	-149.23	Tundra	2009-2009	$0.149e^{0.0593x}$	0.87
Nat_A2	63.88	-149.23	Tundra	2009-2010	$0.149e^{0.0593x}$	0.87
Nat_A3	63.88	-149.23	Tundra	2009-2011	$0.149e^{0.0593x}$	0.87
Nat_AP1	63.88	-149.23	Tundra	2009-2009	$0.149e^{0.0593x}$	0.87
Nat_AP2	63.88	-149.23	Tundra	2009-2010	$0.149e^{0.0593x}$	0.87
Nat_BN1	63.88	-149.23	Tundra	2009-2009	$0.149e^{0.0593x}$	0.87
Nat_BN2	63.88	-149.23	Tundra	2009-2010	$0.149e^{0.0593x}$	0.87
Nat_CB1	63.88	-149.23	Tundra	2009-2009	$0.149e^{0.0593x}$	0.87
Nat_CB2	63.88	-149.23	Tundra	2009-2010	$0.149e^{0.0593x}$	0.87
Nat_EN1	63.88	-149.23	Tundra	2009-2009	$0.149e^{0.0593x}$	0.87
Nat_EN2	63.88	-149.23	Tundra	2009-2010	$0.149e^{0.0593x}$	0.87
Nat_EV1	63.88	-149.23	Tundra	2009-2009	$0.149e^{0.0593x}$	0.87
Nat_EV2	63.88	-149.23	Tundra	2009-2010	$0.149e^{0.0593x}$	0.87
Nat_MS1	63.88	-149.23	Tundra	2009-2009	$0.149e^{0.0593x}$	0.87
Nat_MS2	63.88	-149.23	Tundra	2009-2010	$0.149e^{0.0593x}$	0.87
Nat_OM1	63.88	-149.23	Tundra	2009-2009	$0.149e^{0.0593x}$	0.87
Nat_OM2	63.88	-149.23	Tundra	2009-2010	$0.149e^{0.0593x}$	0.87
Nat_RC1	63.88	-149.23	Tundra	2009-2009	$0.149e^{0.0593x}$	0.87
Nat_RC2	63.88	-149.23	Tundra	2009-2010	$0.149e^{0.0593x}$	0.87
Nat_RS1	63.88	-149.23	Tundra	2009-2009	$0.149e^{0.0593x}$	0.87
Nat_RS2	63.88	-149.23	Tundra	2009-2010	$0.149e^{0.0593x}$	0.87
Nat_VU1	63.88	-149.23	Tundra	2009-2009	$0.149e^{0.0593x}$	0.87
Nat_VU2	63.88	-149.23	Tundra	2009-2010	$0.149e^{0.0593x}$	0.87
Nat_VV1	63.88	-149.23	Tundra	2009-2009	$0.149e^{0.0593x}$	0.87
Nat_VV2	63.88	-149.23	Tundra	2009-2010	$0.149e^{0.0593x}$	0.87
Sha_IG	68.63	-149.57	Tundra	1989-1994	$0.1594e^{0.0434x}$	0.91
Sha_OG	68.63	-149.57	Tundra	1989-1994	$0.1594e^{0.0434x}$	0.91
Upd_BHD	47	-92	Bog	1995-1997	$0.1594e^{0.0434x}$	0.93
Upd_BHM	47	-92	Bog	1995-1997	$0.1594e^{0.0434x}$	0.93
Upd_BHW	47	-92	Bog	1995-1997	$0.1594e^{0.0434x}$	0.93
Upd_BLD	47	-92	Bog	1995-1997	$0.1594e^{0.0434x}$	0.93
Upd_BLM	47	-92	Bog	1995-1997	$0.1594e^{0.0434x}$	0.93
Upd_BLW	47	-92	Bog	1995-1997	$0.1594e^{0.0434x}$	0.93
Upd_FHD	47	-92	Fen	1995-1997	$0.1594e^{0.0434x}$	0.93
Upd_FHM	47	-92	Fen	1995-1997	$0.1594e^{0.0434x}$	0.93
Upd_FHW	47	-92	Fen	1995-1997	$0.1594e^{0.0434x}$	0.93
Upd_FLD	47	-92	Fen	1995-1997	$0.1594e^{0.0434x}$	0.93
Upd_FLM	47	-92	Fen	1995-1997	$0.1594e^{0.0434x}$	0.93

Upd_FLW	47	-92	Fen	1995-1997	$0.1594e^{0.0434x}$	0.93
Wel_BM	47	-88	Bog	1994-1997	$0.1193e^{0.0882x}$	0.92
Wel_BN	47	-89	Bog	1994-1997	$0.1193e^{0.0882x}$	0.92
Wel_BS	47	-88	Bog	1994-1997	$0.1193e^{0.0882x}$	0.92
Wel_FG	47	-89	Fen	1994-1997	$0.1193e^{0.0882x}$	0.92
Wel_FS	47	-88	Fen	1994-1997	$0.1193e^{0.0882x}$	0.92
Wel_GF	47	-88	Fen	1994-1997	$0.1193e^{0.0882x}$	0.92
Wel_MB	47	-89	Bog	1994-1997	$0.1193e^{0.0882x}$	0.92
Wel_NB	47	-88	Bog	1994-1997	$0.1193e^{0.0882x}$	0.92
Wel_SB	47	-89	Bog	1994-1997	$0.1193e^{0.0882x}$	0.92
Wel_SF	47	-89	Fen	1994-1997	$0.1193e^{0.0882x}$	0.92

Supplementary Table 2 Supporting information for synthesized 6 warming experiments to estimate the response of vegetation growth to increasing VPD in the northern peatlands.

ID	Location		Wetland types	Warming Air (°C)	Duration (Years)	Indicators	Refer
	Lat	Lon					
1	68.63	-149.57	Tundra	5.3	6	Above-ground biomass	[1]
2	68.63	-149.57	Tundra	0.8	1	Total plant biomass	[2]
3	68.63	-149.57	Tundra	4.0	4	Total plant biomass	[3]
4	68.35	18.82	Tundra	0.8	3	LAI	[4]
5	41.97	128.12	Tundra	1.0	1	Gross primary production	[5]
6	47.50	-93.48	Bog	2.3	3	Community coverage	[6]

Note: Vegetation growth indicates that the indicators were used to measure vegetation growth in our study.

Supplementary Table 3 Supporting information for 95 FLUXNET 2015 sites.

SITE_ID	LAT	LONG	IGBP	YEAR_START	YEAR_END
AR-Vir	-28.2395	-56.1886	ENF	2009	2012
AU-Ade	-13.0769	131.1178	WSA	2007	2009
AU-ASM	-22.283	133.249	SAV	2010	2014
AU-Cpr	-34.0021	140.5891	SAV	2010	2014
AU-Dry	-15.2588	132.3706	SAV	2008	2014
AU-Emr	-23.8587	148.4746	GRA	2011	2013
AU-Fog	-12.5452	131.3072	WET	2006	2008
AU-Gin	-31.3764	115.7138	WSA	2011	2014
AU-GWW	-30.1913	120.6541	SAV	2013	2014
AU-RDF	-14.5636	132.4776	WSA	2011	2013
AU-Rig	-36.6499	145.5759	GRA	2011	2014
AU-Tum	-35.6566	148.1517	EBF	2001	2014
AU-Wac	-37.4259	145.1878	EBF	2005	2008
AU-Whr	-36.6732	145.0294	EBF	2011	2014
AU-Wom	-37.4222	144.0944	EBF	2010	2014
AU-Ync	-34.9893	146.2907	GRA	2012	2014
BE-Bra	51.3076	4.5198	MF	1996	2014
BR-Sa1	-2.8567	-54.9589	EBF	2002	2011
BR-Sa3	-3.018	-54.9714	EBF	2000	2004
CA-Gro	48.2167	-82.1556	MF	2003	2014
CA-Man	55.8796	-98.4808	ENF	1994	2008
CA-NS1	55.8792	-98.4839	ENF	2001	2005
CA-NS2	55.9058	-98.5247	ENF	2001	2005
CA-NS3	55.9117	-98.3822	ENF	2001	2005
CA-NS4	55.9144	-98.3806	ENF	2002	2005
CA-NS7	56.6358	-99.9483	OSH	2002	2005
CA-Qfo	49.6925	-74.3421	ENF	2003	2010
CG-Tch	-4.2892	11.6564	SAV	2006	2009
CH-Cha	47.2102	8.4104	GRA	2005	2014
CH-Dav	46.8153	9.8559	ENF	1997	2014
CN-Cha	42.4025	128.0958	MF	2003	2005
CN-Dan	30.4978	91.0664	GRA	2004	2005
CN-Du2	42.0467	116.2836	GRA	2006	2008
CN-Qia	26.7414	115.0581	ENF	2003	2005
CZ-BK1	49.5021	18.5369	ENF	2004	2014
CZ-BK2	49.4944	18.5429	GRA	2004	2012
DE-Akm	53.8662	13.6834	WET	2009	2014
DE-Gri	50.95	13.5126	GRA	2004	2014
DE-Obe	50.7867	13.7213	ENF	2008	2014
DE-RuR	50.6219	6.3041	GRA	2011	2014
ES-LgS	37.0979	-2.9658	OSH	2007	2009

FI-Hyy	61.8474	24.2948	ENF	1996	2014
FR-Fon	48.4764	2.7801	DBF	2005	2014
FR-LBr	44.7171	-0.7693	ENF	1996	2008
FR-Pue	43.7413	3.5957	EBF	2000	2014
GF-Guy	5.2788	-52.9249	EBF	2004	2014
GH-Ank	5.2685	-2.6942	EBF	2011	2014
IT-CA1	42.3804	12.0266	DBF	2011	2014
IT-CA3	42.38	12.0222	DBF	2011	2014
IT-Lav	45.9562	11.2813	ENF	2003	2014
IT-MBo	46.0147	11.0458	GRA	2003	2013
IT-Tor	45.8444	7.5781	GRA	2008	2014
JP-MBF	44.3869	142.3186	DBF	2003	2005
JP-SMF	35.2617	137.0788	MF	2002	2006
NL-Hor	52.2403	5.0713	GRA	2004	2011
NL-Loo	52.1666	5.7436	ENF	1996	2014
RU-Fyo	56.4615	32.9221	ENF	1998	2014
RU-Ha1	54.7252	90.0022	GRA	2002	2004
RU-Sam	72.3738	126.4958	GRA	2002	2014
SD-Dem	13.2829	30.4783	SAV	2005	2009
SN-Dhr	15.4028	-15.4322	SAV	2010	2013
US-AR2	36.6358	-99.5975	GRA	2009	2012
US-ARb	35.5497	-98.0402	GRA	2005	2006
US-ARc	35.5465	-98.04	GRA	2005	2006
US-Blo	38.8953	-120.633	ENF	1997	2007
US-Cop	38.09	-109.39	GRA	2001	2007
US-GBT	41.3658	-106.24	ENF	1999	2006
US-GLE	41.3665	-106.24	ENF	2004	2014
US-Goo	34.2547	-89.8735	GRA	2002	2006
US-Ha1	42.5378	-72.1715	DBF	1991	2012
US-LWW	34.9604	-97.9789	GRA	1997	1998
US-Me2	44.4523	-121.557	ENF	2002	2014
US-Me3	44.3154	-121.608	ENF	2004	2009
US-Me4	44.4992	-121.622	ENF	1996	2000
US-Me5	44.4372	-121.567	ENF	2000	2002
US-Me6	44.3233	-121.608	ENF	2010	2014
US-MMS	39.3232	-86.4131	DBF	1999	2014
US-Myb	38.0499	-121.765	WET	2010	2014
US-NR1	40.0329	-105.546	ENF	1998	2014
US-PFa	45.9459	-90.2723	MF	1995	2014
US-SRC	31.9083	-110.84	OSH	2008	2014
US-SRG	31.7894	-110.828	GRA	2008	2014
US-SRM	31.8214	-110.866	WSA	2004	2014
US-Sta	41.3966	-106.802	OSH	2005	2009
US-Syv	46.242	-89.3477	MF	2001	2014

US-Ton	38.4309	-120.966	WSA	2001	2014
US-Tw1	38.1074	-121.647	WET	2012	2014
US-UMB	45.5598	-84.7138	DBF	2000	2014
US-UMd	45.5625	-84.6975	DBF	2007	2014
US-Var	38.4133	-120.951	GRA	2000	2014
US-WCr	45.8059	-90.0799	DBF	1999	2014
US-Whs	31.7438	-110.052	OSH	2007	2014
US-Wi4	46.7393	-91.1663	ENF	2002	2005
US-Wkg	31.7365	-109.942	GRA	2004	2014
ZA-Kru	-25.0197	31.4969	SAV	2000	2013

DBF: deciduous broadleaf forest; EBF: evergreen broadleaf forest; ENF: evergreen needleleaf forest; GRA: grassland; MF: mixed forest; WET: wetland; SAV: savanna; OSH: Open Shrublands; WSA: Woody Savannas; LAT: Latitude; LONG: Longitude; IGBP: International Geosphere Biosphere Programme; MAT: Air temperature; MAP: Precipitation.

Supplementary Table 4 Supporting information for 18 FLUXNET-CH_4 Community Product sites.

SITE_ID	LAT	LONG	CLASSIFICATION	YEAR_START	YEAR_END
CA-SCB	61.3089	-121.298	Bog	2014	2017
DE-Hte	54.21028	12.17611	Fen	2011	2018
DE-SfN	47.80639	11.3275	Bog	2012	2014
DE-Zrk	53.87594	12.88901	Fen	2013	2018
FI-Lom	67.99724	24.20918	Fen	2006	2010
FI-Si2	61.8372	24.1967	Bog	2012	2016
FI-Sii	61.83265	24.19285	Fen	2013	2018
JP-BBY	43.32301	141.8107	Bog	2015	2018
SE-Deg	64.18203	19.55654	Fen	2014	2018
SE-St1	68.35415	19.05033	Fen	2012	2014
US-A03	70.49533	-149.882	Wet tundra	2015	2018
US-A10	71.3242	-156.615	Wet tundra	2012	2018
US-BZB	64.69555	-148.321	Bog	2014	2016
US-BZF	64.70373	-148.313	Fen	2014	2016
US-Ivo	68.4865	-155.75	Wet tundra	2013	2016
US-Los	46.0827	-89.9792	Fen	2014	2018
US-Uaf	64.86627	-147.856	Bog	2011	2018
RU-Cok	70.82914	147.4943	Wet tundra	2008	2016

LAT: Latitude; LONG: Longitude

Supplementary Table 5 Supporting information for synthesized 5 studies to estimate the differences in evapotranspiration between moss and vascular plant in the northern peatlands.

ID	Location		Main moss species	Main vascular plant	Observation types	References
	Lat	Lon				
1	NA	NA	*Tortella tortuosa*, *Syntrichia ruralis*	*Sedum album, Artemisia campestris, Thymus Serpyllum, Helianthemum nummularium, Poa alpina*	Field observations	[7]
2	45.10	141.70	*Sphagnum fuscum, Sphagnum magellanicum*	*Sasa palmate, Moliniopsis japonica, Myrica gale, tomentosa, Ilex crenata*	Field observations	[8]
3	52.70	6.42	*Sphagnum magellanicum*	*Vaccinium oxycoccus L., Erica tetralix L., Eriophorum angustifolium*	Field observations	[9]
4	45.10	141.70	*Sphagnum papillosum. Sphagnum magellanicum*	*Myrica gale, Ilex crenata*	Eddy covariance towers	[10]
5	54.78~55.90	-113.53~ -112.47	Open moss-dominated poor fen	Open sedge-dominated extreme-rich fen, Wooded moderately rich fen	Eddy covariance towers	[11]

Supplementary Table 6 Data sources of satellite, eddy-covariance flux, meta-analysis.

Datasets	Data sources
FLUXNET 2015	https://fluxnet.org/data/fluxnet2015-dataset/
FLUXNET-CH_4 Community	https://fluxnet.org/data/fluxnet-ch4-community-product/
FLUXCOM GPP	https://www.bgc-jena.mpg.de/geodb/projects/Home.php
VPM GPP	https://data.tpdc.ac.cn/en/data/582663f5-3be7-4f26-bc45-b56a3c4fc3b7/
GOSIF GPP	http://data.globalecology.unh.edu/data/GOSIF-GPP_v2/
MODIS ET/GPP	https://modis.gsfc.nasa.gov/data/dataprod/mod16.php
CRU TS 4.04 (land)	http://climexp.knmi.nl/selectfield_obs2.cgi?id=someone@somewhere
ERA5-Land	https://cds.climate.copernicus.eu/#!/home
PLM-v2	https://data.tpdc.ac.cn/zh-hans/data/48c16a8d-d307-4973-abab-972e9449627c/
GSD	https://data.tpdc.ac.cn/en/data/2e46eb77-3ca2-4b90-9a42-fd49f10630d4/
TerraClimate	https://www.climatologylab.org/terraclimate.html
AI_PET_v3	https://figshare.com/articles/dataset/Global_Aridity_Index_and_Potential_Evapotranspiration_ET0_Climate_Database_v2/7504448/6
SSM	http://doi.org/10.5281/zenodo.7503012
PEATMAP	https://archive.researchdata.leeds.ac.uk/251/
Peat-ML	https://zenodo.org/record/5794336
GLASS-GLC	https://doi.pangaea.de/10.1594/PANGAEA.898096

References

1. Shaver G*, et al.* Biomass and CO2 flux in wet sedge tundras: responses to nutrients, temperature, and light. *Ecological Monographs* **68**, 75-97 (1998).
2. Grogan P, Chapin Iii F. Initial effects of experimental warming on above-and belowground components of net ecosystem CO2 exchange in arctic tundra. *Oecologia* **125**, 512-520 (2000).
3. Hobbie SE, Chapin III FSJE. The response of tundra plant biomass, aboveground production, nitrogen, and CO2 flux to experimental warming. *Ecology* **79**, 1526-1544 (1998).
4. Sundqvist MK*, et al.* Responses of tundra plant community carbon flux to experimental warming, dominant species removal and elevation. *Functional Ecology* **34**, 1497-1506 (2020).
5. Zhou Y*, et al.* Effects of experimental warming on growing season temperature and carbon exchange in an alpine tundra ecosystem. *Russian Journal of Ecology* **50**, 474-481 (2019).
6. Norby RJ, Childs J, Hanson PJ, Warren JMJE, Evolution. Rapid loss of an ecosystem engineer: Sphagnum decline in an experimentally warmed bog. *Ecology and Evolution* **9**, 12571-12585 (2019).
7. Sand-Jensen K, Hammer KJ, Madsen-Østerbye M, Dencker T, Kragh T. Positive interactions between moss cushions and vascular plant cover improve water economy on Öland's alvar, Sweden. *Botany* **93**, 141-150 (2015).
8. Takagi K, Tsuboya T, Takahashi H, Inoue T. Effect of the invasion of vascular plants on heat and water balance in the Sarobetsu mire, northern Japan. *Wetlands* **19**, 246-254 (1999).
9. Heijmans MM, Arp WJ, Berendse F. Effects of elevated CO2 and vascular plants on evapotranspiration in bog vegetation. *Global Change Biology* **7**, 817-827 (2001).
10. Hirano T, Yamada H, Takada M, Fujimura Y, Fujita H, Takahashi H. Effects of the expansion of vascular plants in Sphagnum-dominated bog on evapotranspiration. *Agricultural Forest Meteorology* **220**, 90-100 (2016).
11. Humphreys ER*, et al.* Summer carbon dioxide and water vapor fluxes across a range of northern peatlands. *Journal of Geophysical Research: Biogeosciences* **111**, 2273-2291 (2006).
