# Supplementary material for: Warming-induced vapor pressure deficit suppression of vegetation growth diminished in northern peatlands
Source: Nat Commun. 2023 Nov 30;14:7885. doi: 10.1038/s41467-023-42932-w (PMC10689446; doi:10.1038/s41467-023-42932-w)
Supplement: Supplementary file 2 — Reporting Summary [file 41467_2023_42932_MOESM2_ESM.pdf]

nature portfolio

Corresponding author(s): Changchun Song, Xiaofeng Xu, Xianwei Wang

Last updated by author(s): Oct 24, 2023

Reporting Summary

Nature Portfolio wishes to improve the reproducibility of the work that we publish. This form provides structure for consistency and transparency in reporting. For further information on Nature Portfolio policies, see our Editorial Policies and the Editorial Policy Checklist.

Statistics

For all statistical analyses, confirm that the following items are present in the figure legend, table legend, main text, or Methods section.

n/a	Confirmed	
☐	☒	The exact sample size (n) for each experimental group/condition, given as a discrete number and unit of measurement
☐	☒	A statement on whether measurements were taken from distinct samples or whether the same sample was measured repeatedly
☐	☒	The statistical test(s) used AND whether they are one- or two-sided<br>*Only common tests should be described solely by name; describe more complex techniques in the Methods section.*
☒	☐	A description of all covariates tested
☐	☒	A description of any assumptions or corrections, such as tests of normality and adjustment for multiple comparisons
☐	☒	A full description of the statistical parameters including central tendency (e.g. means) or other basic estimates (e.g. regression coefficient) AND variation (e.g. standard deviation) or associated estimates of uncertainty (e.g. confidence intervals)
☐	☒	For null hypothesis testing, the test statistic (e.g. F, t, r) with confidence intervals, effect sizes, degrees of freedom and P value noted<br>*Give P values as exact values whenever suitable.*
☒	☐	For Bayesian analysis, information on the choice of priors and Markov chain Monte Carlo settings
☒	☐	For hierarchical and complex designs, identification of the appropriate level for tests and full reporting of outcomes
☒	☐	Estimates of effect sizes (e.g. Cohen's d, Pearson's r), indicating how they were calculated

Our web collection on statistics for biologists contains articles on many of the points above.

Software and code

Policy information about availability of computer code

Data collection	GetData Graph Digitizer 2.24
Data analysis	The data in this study were analyzed with publicly available tool packages in R 4.3.0. All the scripts are available from https://figshare.com/s/a1b39bfc3a2077cc0515.

For manuscripts utilizing custom algorithms or software that are central to the research but not yet described in published literature, software must be made available to editors and reviewers. We strongly encourage code deposition in a community repository (e.g. GitHub). See the Nature Portfolio guidelines for submitting code & software for further information.

Data

Policy information about availability of data

All manuscripts must include a data availability statement. This statement should provide the following information, where applicable:

- Accession codes, unique identifiers, or web links for publicly available datasets
- A description of any restrictions on data availability
- For clinical datasets or third party data, please ensure that the statement adheres to our policy

The datasets supporting this study’s findings are available from https://figshare.com/s/a1b39bfc3a2077cc0515. FLUXNET 2015: https://fluxnet.org/data/fluxnet2015-dataset/; FLUXNET-CH4 Community: https://fluxnet.org/data/fluxnet-ch4-community-product/; FLUXCOM GPP: https://www.bgc-jena.mpg.de/geodb/projects/Home.php; VPM GPP: https://data.tpdc.ac.cn/en/data/582663f5-3be7-4f26-bc45-b56a3c4fc3b7/; GOSIF GPP: http://data.globalecology.unh.edu/data/GOSIF-GPP_v2/; MODIS ET/GPP: https://modis.gsfc.nasa.gov/data/dataprod/mod16.php; CRU TS 4.04 (land): http://climexp.knmi.nl/selectfield_obs2.cgi?

id=someone@somewhere; ERA5-Land: https://cds.climate.copernicus.eu/#!/home; PLM-v2: https://data.tpdc.ac.cn/zh-hans/data/48c16a8d-d307-4973-abab-972e9449627c/; GSD: https://data.tpdc.ac.cn/en/data/2e46eb77-3ca2-4b90-9a42-fd49f10630d4/; TerraClimate: https://www.climatologylab.org/terraclimate.html; AI_PET_v3: https://figshare.com/articles/dataset/Global_Aridity_Index_and_Potential_Evapotranspiration_ET0_Climate_Database_v2/7504448/6; SSM: http://doi.org/10.5281/zenodo.7503012; PEATMAP: https://archive.researchdata.leeds.ac.uk/251/; Peat-ML: https://zenodo.org/record/5794336; GLASS-GLC : https://doi.pangaea.de/10.1594/PANGAEA.898096.

Research involving human participants, their data, or biological material

Policy information about studies with human participants or human data. See also policy information about sex, gender (identity/presentation), and sexual orientation and race, ethnicity and racism.

Reporting on sex and gender	This is not relevant to this study.
Reporting on race, ethnicity, or other socially relevant groupings	This is not relevant to this study.
Population characteristics	This is not relevant to this study.
Recruitment	This is not relevant to this study.
Ethics oversight	This is not relevant to this study.

Note that full information on the approval of the study protocol must also be provided in the manuscript.

Field-specific reporting

Please select the one below that is the best fit for your research. If you are not sure, read the appropriate sections before making your selection.

☐ Life sciences ☐ Behavioural & social sciences ☒ Ecological, evolutionary & environmental sciences

For a reference copy of the document with all sections, see nature.com/documents/nr-reporting-summary-flat.pdf

Ecological, evolutionary & environmental sciences study design

All studies must disclose on these points even when the disclosure is negative.

Study description	We compiled multisource datasets of in situ observations, a multisite synthesis (78 sites), eddy covariance flux towers (18 sites from FLUXNET-CH4 Community Product and 95 sites from FLUXNET2015), and regional-scale remote sensing products to explore the effects of increasing VPD and investigate their underlying mechanisms in northern peatlands.
Research sample	For the warming experiment in Mohe, Ta (control, mean ± SE, 14.44±0.99℃° C℃℃; warming, 18.28±0.94℃° C℃℃), RH (control,71.93±1.21%; warming,70.88±1.17%), and VPD (control,4.71±0.28hPa; warming, 6.21±0.33hPa) were observed by a microclimate instrument (Detailed information in Data collection). Ta (control, 14.12±1.01℃℃° C℃℃; warming, 16.77±1.20℃° C ℃℃), RH (control,75.76±4.40%; warming,70.88±1.17%), and VPD (control,4.28±0.84hPa; warming, 6.86±1.75hPa) in synthesized warming experiment sites were collected in a meta-analysis. We also measured the Gc of Vaccinium uliginosum in Mohe and collected NPP in synthesized warming experiment sites. In addition, we collected 16 comparative data pairs from 5 studies (1.19 ±0.16℃mm/day vs. 1.62±0.16mm/day℃℃) to compare ET between vascular plants and mosses .The flux tower-based GPP, latent heat flux (LE, W m−2), sensible heat flux (H, W m−2), and environmental variables of Ta, VPD, precipitation, shortwave radiation, wind speed (m s−1), friction velocity (u8, unitless), and atmospheric pressure were obtained from the global eddy-covariance flux dataset, FLUXNET2015 and FLUXNET-CH4 Community Product. Monthly solar radiation was derived from the reanalysis products of ERA5-Land; The monthly wind speed was obtained from TerraClimate; Monthly Ta, precipitation, and AVP were obtained from CRU 4.04 datasets; Monthly RH was the ratio between AVP and SVP. The ratio of precipitation to potential evapotranspiration was defined as AI (arid and semiarid and dry subhumid regions, AI < 0.65; humid, AI ≥ 0.65) (Global-AI_PET_v3); GPP derived from VPM GPP, GOSIF GPP and FLUXCOM GPP; ET derived from MODIS datasets; Et was estimated by a coupled diagnostic biophysical model (PML-v2). The sources of flux tower-based datasets and satellite-derived datasets were the same in the Data section.
Sampling strategy	Random forest models were applied to parse the sensitivity of the VPD effects to plant traits and environmental factors using the randomForest function in the randomForest package. Sixty percent of the data were used to train the models, and the remaining 40% were used for validation. We used to sample () function and set.seed () function in R 4.3.0 to determine the sample using in the analysis of randomForest. The size of train sample and validation sample was greater than 1000, meeting analytical needs.
Data collection	At the Mohe site, TC and TN were detected using a TC-TN analyzer (Shimadzu, Tokyo, Japan). Ta, RH and VPD were measured by a portable temperature and humidity sensor (5TM, METER Group Inc) attached to a data logger (EM50/G, METER Group Inc.). Gc of Vaccinium uliginosum was measured by an AP4 Porometer (AP4, Delta-t, UK). Multi-site synthesis of warming experiments and moss ET vs. vascular plants ET were collected by searching for all peer-reviewed publications (1990-2022) investigating the response of wetland GHG emissions to warming, using Google Scholar (https://scholar.google.com), Web of Science (https://www.webofscience.com/) and China National Knowledge Infrastructure Databases (http://www.cnki.net) and a recent meta-analysis. Other datasets were the same in the Data section. These datasets were mainly collected by N.C and Y.Z.
Timing and spatial scale	Ta, RH and VPD were recorded at 30-min intervals. We installed the two sensors in each treatment (N = 2 per treatment) and took their average. In the peak growing season, diurnal dynamic measurements of Gc (9 AM, 14 PM, 17 PM; China Standard Time) were

	conducted on August 9 and 18, 2021.
Data exclusions	No data were excluded from the analyses.
Reproducibility	The data and variables currently used in our study may be the most comprehensive dataset we could find at this time. In order to guarantee reproducibility, we described in detail the field warming experiment in Mohe site. We used the multi-site synthesis of warming experiments in our study from a recent meta-analysis that used the funnel plot method and Egger's regression asymmetry test. The regional-scale analyses were provided the code and data in https://figshare.com/s/a1b39bfc3a2077cc0515.
Randomization	For the Gc, we randomly measured five leaves of Vaccinium uliginosum at different heights of its canopy in each mesocosm (square plastic barrel) under the control and warming treatments in Mohe. For the randomForest analyses, sample () function and set.seed () function in R 4.3.0 were used to random sampling.
Blinding	Blinding is not relevant to our study, because we collected as much and comprehensive data as possible in our study.

Did the study involve field work? ☒ Yes ☐ No

Field work, collection and transport

Field conditions	The warming experiment in Mohe site is characterized by a humid monsoon climate in a cold temperate zone with mean annual temperature and precipitation of −3.9 °C and 450 mm, respectively. The growing season lasts for c. 120 days, from mid-May to mid-September. Four common native plant species in the plant community are Sphagnum palustre, Vaccinium uliginosum, Ledum palustre, and Carex globularis.
Location	The warming experiment in Mohe site was conducted in a peatland in the northern Greater Hinggan Mountains (Tuqiang Forestry Bureau in Mohe city, Heilongjiang province; 52.93°N, 122.83°E)
Access & import/export	Our study is in the Tujung Forestry District, we have a long-term relationship, and our experiments and sampling are in accordance with the regulations of the Tujung Forestry Department. Therefore, this is not relevant to our study.
Disturbance	Individual species attributes as well as soil attributes may affect the results of warming experiments in Mohe. To guarantee homogeneities in the plants and soils, we implemented the following manipulations. (1) plants with relatively uniform height and coverage were selected from nearby natural peatlands. On the same day, we cleaned the selected plants by removing soil particles under running water and then weighed these plants. We found that the wet weight of the selected plants showed no significant differences between warming (mean ± 1 se, 476.2 ± 21.2g m−2) and control (451.6 ± 23.1g m−2) treatments (p = 0.477). (2) the square plastic barrels were filled with peat soils (thickness of 30–35cm) and manually collected in the peatlands from which the plants were sourced. Soil total carbon (TC, 346.0 ± 9.1 vs. 336.5 ± 4.0mg g−1, p = 0.393) and total nitrogen (TN, 27.8 ± 0.5 vs. 28.9 ± 1.7mg g−1, p = 0.578) showed insignificant differences between warming and control treatments. Additionally, the water in the control treatment was entirely supplied by precipitation. For the warming treatment, precipitation was collected in a container and then evenly sprinkled on each square plastic barrel in the transparent greenhouse after precipitation events. Mean soil moisture content (SMC, %) in the warming treatment decreased insignificantly by 2.0% (33.3 ± 0.5 vs. 31.3 ± 0.7%, p = 0.073) compared to the control treatment in the growing season of 2021.

Reporting for specific materials, systems and methods

We require information from authors about some types of materials, experimental systems and methods used in many studies. Here, indicate whether each material, system or method listed is relevant to your study. If you are not sure if a list item applies to your research, read the appropriate section before selecting a response.

Materials & experimental systems

n/a	Involved in the study
☒	☐ Antibodies
☒	☐ Eukaryotic cell lines
☒	☐ Palaeontology and archaeology
☒	☐ Animals and other organisms
☒	☐ Clinical data
☒	☐ Dual use research of concern
☒	☐ Plants

Methods

n/a	Involved in the study
☒	☐ ChIP-seq
☒	☐ Flow cytometry
☒	☐ MRI-based neuroimaging

Plants

Seed stocks	This is not relevant to this study.
Novel plant genotypes	This is not relevant to this study.
Authentication	This is not relevant to this study.
